# A Perspective on Perovskite Solar Cells: Emergence, Progress, and Commercialization

**DOI:** 10.3389/fchem.2022.802890

**Published:** 2022-04-11

**Authors:** Pengyu Zhang, Menglin Li, Wen-Cheng Chen

**Affiliations:** ^1^ School of Materials Science and Engineering, Beijing Institute of Technology, Beijing, China; ^2^ Beijing JAYU New Energy Technology Development Co., Ltd., JAYU Group, Beijing, China; ^3^ School of Chemical Engineering and Light Industry, Guangdong University of Technology, Guangzhou, China

**Keywords:** perovskite, solar cells, composition engineering, process engineering, interfacial engineering, industrial progress, commercialization

## Abstract

With rapid progress in light-to-electric conversion efficiencies, perovskite solar cells (PSCs) have exhibited great potential as next-generation low-cost, efficient photovoltaic technology. In this perspective, we briefly review the development of PSCs from discovery to laboratory research to commercializing progress. The past several decades have witnessed great achievement in device efficiency and stability due to tremendous research efforts on compositional, process, and interfacial engineering. Regarding commercial applications, we expound the merits and disadvantages of PSCs compared to the existing silicon photovoltaic technologies. Although PSCs promise solution processability and low manufacturing cost, their limited stability and element toxicity should to be addressed on the path to commercialization. Finally, we provide future perspectives on commercialization of PSCs in the photovoltaic marketplace. It is suggested that PSCs will be more promising in low-cost modules and tandem configurations.

## Graphical Abstract

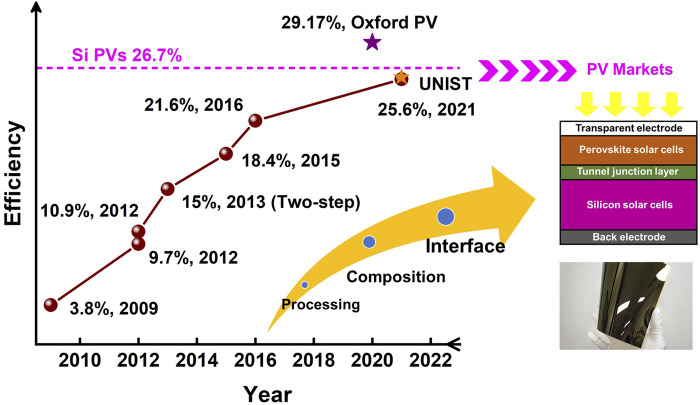

## Emergence of Perovskite Solar Cells

Metal halide perovskites (MHPs) have attracted intensive attention as promising photovoltaic materials during the last few decades. The term of “Perovskite” is employed to describe a class of materials with the same crystal structure as the mineral calcium titanate (CaTiO_3_), which was firstly discovered in the Ural Mountains of Russia in 1839 and named after Lev Perovski, a renowned Russian mineralogist (Chakhmouradian and Woodward, 2014). Now, MHPs have developed into a broad range of materials with the general formula ABX_3_, where A and B are monovalent and divalent cations, respectively and X stands for anions. Besides, MHPs have been widely used as active materials in various optical and electronic applications, such as photovoltaic devices (PV) (Kojima et al., 2009; Kim et al., 2012; Lee et al., 2012; Jeong et al., 2021), light-emitting diode (Cho et al., 2015; Cao et al., 2018; Lin et al., 2018), photodetector (Li et al., 2020a; Li et al., 2020b), laser (Sutherland and Sargent, 2016; Wei et al., 2019), sensor (Zaza et al., 2019; George et al., 2020), biomedicine (Fang et al., 2021; Zhang et al., 2021; Fang et al., 2022) etc.

**FIGURE 1:**
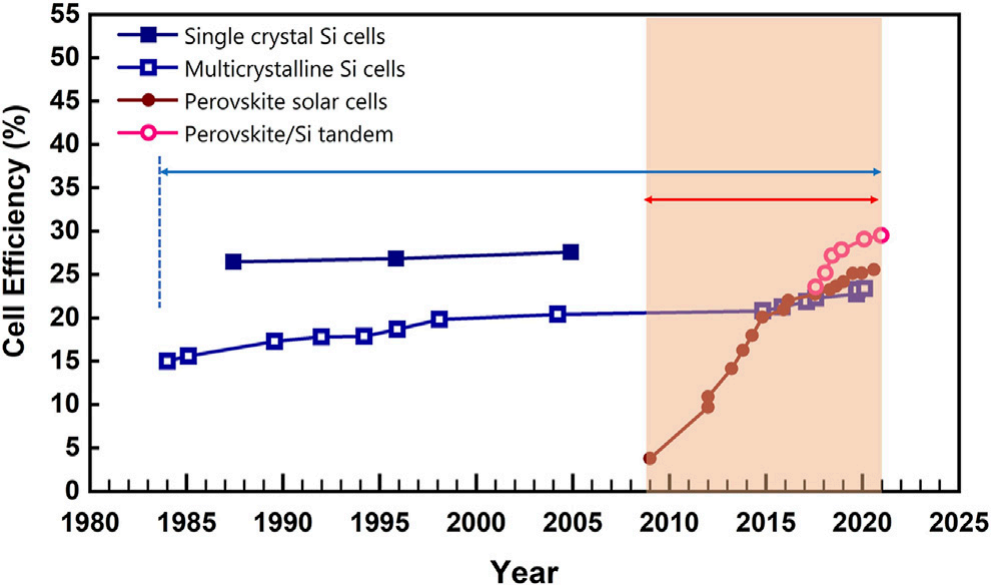
Progress of cell efficiency in single crystal and multicrystalline Si cells, perovskite solar cells, perovskite/Si tandem according to Best Research-Cell Efficiency chart from National Renewable Energy Laboratory (NREL, 2020).

The first demonstration of the photovoltaic effect on perovskite materials dates back to 2009 by Miyaska and his co-works, but the power conversion efficiency (PCE) was only 3.8% (Kojima et al., 2009). Although the performance of perovskite solar cells (PSCs) was low at that time, the strong optical absorption of perovskite materials attracted widespread attention in academic circles. However, these cells suffered from rapid degradation of perovskite materials in the liquid electrolyte. In 2012, important breakthroughs in PSCs were realized by (Kim et al., 2012; Lee et al., 2012). In these works, all-solid device configurations were reported with Spiro-MeOTAD [2,2′,7,7′-tetrakis (*N,N*-di-p-methoxyphenylamine)-9,9′-spirobifluorene] as the hole transport layer to solve the instability problem in liquid electrolytes. The PCEs of about 10% have been reported with improved operation stability. Now, the most recent world record for single-junction PSCs has reached 25.6%, claimed by researchers at South Korea’s Ulsan National Institute of Science and Technology (UNIST) (Jeong et al., 2021). As demonstrated in Figure 1, the laboratory efficiency of PSCs is comparable to that of the first-generation monocrystalline silicon solar cell that takes about 40 years for this level.

In addition, perovskites have been demonstrated as promising candidates in multi-junction cells due their easily tunable bandgap through constituents (Al-Ashouri et al., 2020; Chen et al., 2021; Chen et al., 2022; Lin et al., 2022; Qin et al., 2022). The state-of-the-art perovskite-on-silicon tandem solar cell has achieved a PCE of 29.52% in Oxford PV on approximately 30 × 30 cm^2^ device area (Al-Ashouri et al., 2020). More recently, the all-perovskite tandem solar cell with a certified efficiency of 26.4% has also been reported by (Lin et al., 2022).

Perovskite materials are excellent light absorbers with outstanding optoelectronic properties such as high light absorption coefficient, long carrier diffusion length (exceeding 1 mm), low non-radiative recombination loss and high defect tolerance (Stranks et al., 2013; Xing et al., 2013; Eperon et al., 2014; Green et al., 2014). The theoretical limit of single-junction PSCs is about 33%, and that of tandem solar cells can reach over 40%. Along with ease of accessibility through low-cost solution processes, there is no doubt that PSCs have become one of the most promising photovoltaic technologies. Since reported in 2009, it was rated as one of the ten scientific breakthroughs by *Science*. Next year, it was elected as one of the most anticipated scientific and technological breakthroughs by *Nature*. In the World Economic Forum of 2016, PSCs were honored as one of the top 10 emerging technologies that would break the limitations of silicon-based photovoltaics. In a world, perovskite materials have become a hot topic in both academic and industrial fields.

## Research Progress

**FIGURE 2:**
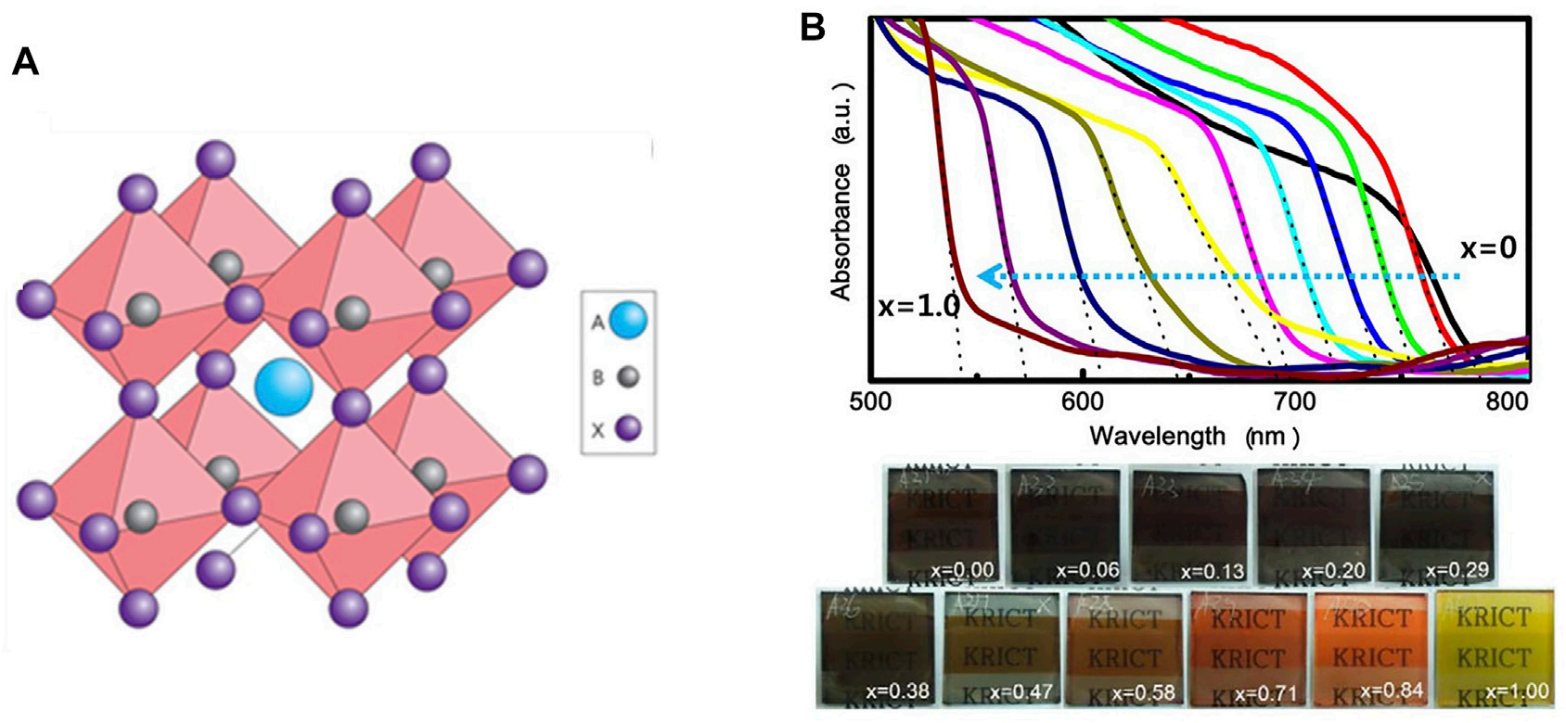
**(A)** Cubic perovskite structure with ABX_3_. Adapted from Green et al. (2014) with permission from Nature springer. **(B)** UV-vis absorption spectra of PSCs with mixing halide ions and corresponding colorful films. Adapted form Noh et al. (2013) with permission from American Chemical Society.

Since reported in 2009, intensive research efforts have been made to improve the efficiency of PSCs, including compositional, process and interfacial engineering. Perovskite photovoltaic materials are ionic compounds in the general formula of ABX_3_, as shown in Figure 2A. The crystallographic structure can be deduced by the Goldschmidt tolerance factor ( $\text{t}=\left(R_{A}+R_{X}\right)/\sqrt{2}\left(R_{B}+R_{X}\right)$ ) and the octahedral factor ( $\text{μ}=R_{B}/R_{X}$ ) (Li et al., 2008). For applications in solar cells, MHPs commonly are obtained with t and *µ* in the range of 0.81–1.1 and 0.44–0.9, respectively. Despite these constraints, a broad range of ions enables good results. For example, cation A has methylammonium MA^+^, formamidinium FA^+^ and cesium (Cs+) and other long-chain organic cations, metallic cation B has universally been Pb^2+^ or Sn^2+^ and anion X is halide ions (Cl^−^, Br^−^, I^−^) or Pseudo-halide ions.

The optoelectronic properties of MHPs are strongly correlated with ion species. Firstly, the bandgap can be continuously tuned in the range of almost the whole visible spectrum by X-site halide mixing (Noh et al., 2013; Eperon et al., 2014). As shown in Figure 2B, the absorption edge moves from 786 to 544 nm with the substitution of I with Br ions, corresponding to the bandgap increasing from 1.58 to 2.28 eV. The bandgap engineering by halide ions provides an effective strategy to design wide-bandgap perovskites into tandem solar cells. Another significant ion substitution is replacing the MA^+^ by FA^+^. Due to the larger ion size of FA^+^, the replacement expands the perovskite crystal and shortens the Pb-I bond distance, resulting in the reduced bandgap of 1.47 eV. The use of FA^+^ as the A-site cation in MHPs further increases photocurrents. Unfortunately, phase stability of FAPbI_3_ still depends on the following chemical management according to Goldschmidt rule. At the ambient conditions, the photoactive α-FAPbI_3_ is unstable and easily transforms into the yellow *δ* phase, which seriously deteriorates the device performance. To solve this problem, new strategies are needed for stabilizing the α-phase in FA-based PSCs. Seok and co-workers firstly stabilized α-phase FAPbI_3_ by incorporation of MAPbBr_3_ and achieved a PCE of more than 18% PCE (Jeon et al., 2015). Later on, A site cation was further optimized as a “cation cascade” by mixing FA^+^, MA^+^ and Rb^+^ to eliminate the residual δ-phase by Saliba and his co-workers (Saliba et al., 2016). By this method, FA-based PSCs can deliver a stabilized PCE of over 21% and a long shelf lifetime of over 1,000 h. However, the use of MA^+^, Rb^+^ and Br^−^ into FAPbI_3_ perovskites can inevitably widen the bandgap, limiting the device performance. Instead, an anion engineering was introduced to stabilize pure phase FAPbI_3_ with the pseudo-halide anion (HCOO^−^) passivating the I- vacancy defects. The resulting solar cells have world-record efficiency of 25.6% and long-term stability over 450 h (Jeong et al., 2021).

The prerequisite for high-efficiency device is to fabricate high-quality perovskite films with a flat and compact morphology. To date, numerous processing techniques have been developed to prepare high-quality perovskite films, including vacuum deposition, one-step solution deposition (antisolvent dripping, solvent-assisted annealing, and precursor engineering), two-step sequential deposition and vapor-assisted deposition. Among these methods, solution processes are promising with low temperature and cost, compatible with flexible electronics. Furthermore, scaling-up methods (slot-die coating, roll-to-roll, and inkjet printing) have also been developed for the industrial manufacturing of PSCs in the future.

### Vacuum Deposition

**FIGURE 3:**
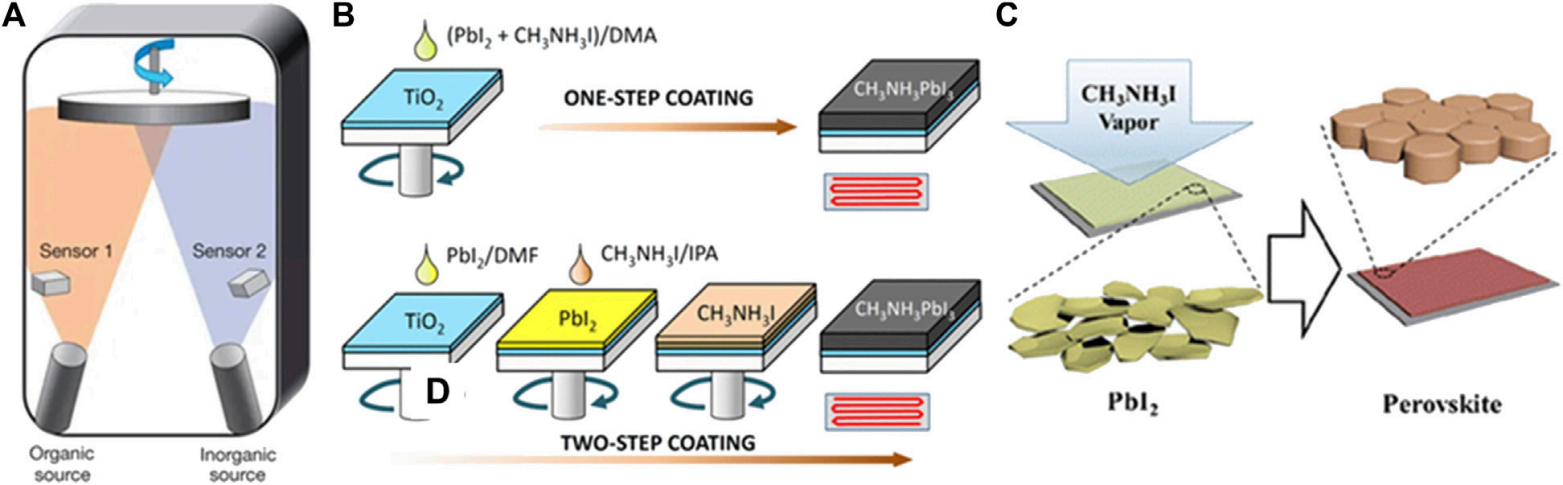
**(A)** Dual-source thermal evaporation. Adapted from Liu et al. (2013) with permission from Nature springer. **(B)** One-step and two-step solution process for perovskite film growth. Adapted from Im et al. (2014) with permission from Nature springer. **(C)** Perovskite film formation through vapor-assisted deposition. Reproduced from Chen et al. (2014) with permission from American Chemical Society.

The vacuum deposition method was firstly reported by Mitzi and co-workers, in which organic-inorganic compounds were rapidly heated to sublimate, then deposited on the substrate (Mitzi et al., 1999). This technique allows facile control over the film composition and thickness with high reproducibility. With the rapid development of PSCs, Snaith et al. employed dual-source co-evaporation to deposit a uniform MAPbI_3-x_Cl_x_ perovskite layer and a PCE of 15% was reported (Figure 3A) (Liu et al., 2013). The perovskite films fabricated by vacuum deposition were proven to be high-quality with excellent film uniformity. Although the vacuum deposition requires expensive equipment, it still holds the advantages of fabricating large-area films without using toxic solvents. This makes it very promising in the production of the perovskite module. Recently, vacuum deposition has witnessed further progress in the perovskites materials including mixed halide (Longo et al., 2018), narrow-bandgap (Igual-Muñoz et al., 2020), and formamidine based perovskite solar cells (Chiang et al., 2020; Feng et al., 2021). The champion efficiency of 21.32% and 18.13% has been reported on the small-size cell (Feng et al., 2021) and mini-modules (effective device area 21 cm^2^) (Li J. et al., 2020), respectively.

### One-Step Solution Deposition

One-step solution deposition is a commonly used method for fabricating perovskite films in labs. In this method, the precursor solutions are prepared by mixing precursors in solvents such as N, N-dimethylformamide (DMF), then spin-coated on the substrates, followed by thermal annealing to initialize the crystallization (Im et al., 2014). To obtain flat and compact films without pin-holes, antisolvents are usually used to induce homogenous nucleation, for example, toluene (Jeon et al., 2014), chlorobenzene (Bi et al., 2016), or diethyl ether (Li et al., 2017; Gao et al., 2018). One-step deposition is simple to conduct in labs and antisolvents can serve as the carriers for Lewis acid and base additives, which has been demonstrated as an effective strategy for defect passivation. For example, Wu et al. achieved highly efficient inverted PSCs with a perovskite-fullerene graded heterojunction formed by dripping the antisolvent containing fullerenes (Wu et al., 2016). Subsequently, conjugated polymers dissolved in antisolvents were introduced in perovskite films to reduce trap states and non-radiative recombination loss (Rajagopal et al., 2017; Qin et al., 2018; Yang et al., 2018; Chen et al., 2019a; Wu et al., 2019).

### Two-Step Solution Deposition

Besides one-step solution deposition, two-step sequential deposition is another commonly used method to obtain perovskite films with improved reproducibility (Burschka et al., 2013; Im et al., 2014). As firstly reported by Burschka et al., PbI_2_ was spin-coated on the substrates followed by sequential deposition of MAI, then the formation of the perovskite was accomplished by thermal annealing, as depicted in Figure 3B. Compared with one-step solution deposition, two-step deposition has better control over the film morphology by relieving effects from the surrounding environment. However, the conversion of PbI_2_ to the perovskite remains a major challenge to achieve highly efficient and stable devices (Zhang et al., 2015; Chen, 2017; Chiang et al., 2017; Xu et al., 2019). The residual PbI_2_ at the interfaces was suggested to impede the carrier transportation and induce severe degradation PSCs (Jiang et al., 2016).

### Vapor-Assisted Deposition

This method was firstly proposed by Chen et al. in which *in-situ* reaction between the as-deposited PbI_2_ (solid) and MAI vapor was adopted to obtain perovskite films with full coverage and smooth surface, as schematically illustrated in Figure 3C (Chen et al., 2014). The vapor-assisted process is a facile low-temperature method with the slowdown in nucleation and film growth, which delivers increasing grain size and high reproducibility. In addition, vapor-assisted deposition provides the possibility of growing high-quality perovskites on curvature or textured substrates. Zou et al. demonstrated a high-quality perovskite film grown on a fibrous substrate by the improved vapor-assisted deposition and the fiber-shaped perovskite solar cell with a PCE of 10.79% was obtained (Dong et al., 2019).

For commercializing PSCs, the large-scale process method has attracted more attention in recent years. With the development of large-area coating methods, including blade, slot-die and spray coating, the roll-roll (R2R) printed process has been suggested to realize the upscaling of PSCs with low cost and high-throughput (Yang et al., 2021). At first, a sequential slot-die method was developed to fabricate large-area PSCs with a PCE up to 11.96% (Hwang et al., 2015). The process was demonstrated to be incorporated into R2R printing. During the past few years, some researchers tried to produce PSCs by the R2R printed process, but only partial processes were reported at laboratory scale (Dou et al., 2018; Galagan et al., 2018; Kim J. E. et al., 2019; Kim Y. Y. et al., 2019). More recently, Kim et al. demonstrated the fully R2R manufacturing of PSCs with PCEs of 23.5% and 19.1% on planar and flexible substrates, respectively. This work suggested the gravure R2R printed process as a promising method to upscaling PSCs in the near future (Kim et al., 2020; Yang et al., 2021).

Besides compositional and process engineering, interfacial engineering has also contributed to the rapid progress in the efficiency and stability of PSCs. Interfacial engineering mainly focuses on charge transport layers (CTLs) together with interfacial modifications. CTLs play important roles in PSCs, which can not only promote the photogenerated carrier extraction but also suppress the non-radiative recombination. An effective CTL should meet the following requirements: first, matched energy level for the charge extraction from perovskite layer; second, high transparency to avoid energy loss of solar light; and finally, high hole/electron mobility, and the blocking ability of the counter carrier (Ouyang et al., 2019).

Mesoporous TiO_2_ and small molecule Spiro-MeOTAD were used as effective electron transport layer (ETL) and hole transport layer (HTL) in the pioneering works (Kim et al., 2012; Lee et al., 2012). Significantly, the use of Spiro-MeOTAD solved the corrosive problem of liquid electrolytes, making all-solid cells. However, Spiro-MeOTAD remains far inferior to commercial photovoltaic cells. Under continuous illumination, doped lithium salt tends to cause aggregation of Spiro-MeOTAD, leading to a rapid decrease in the device efficiency (Abate et al., 2013). On the other side, mesoporous TiO2 induces fast decay due to exposed oxygen vacancies under ultraviolet light (Leijtens et al., 2013). Therefore, more stable CTLs with effective charge extraction are still required to address the stability issue. Up till now, a great number of CTLs have been studied to deliver more efficient and stable PSCs, including small molecules, polymers and inorganic materials. In terms of long-term operational stability, inorganic materials would be more suitable for the commercialization of PSCs due to chemical inertness and low cost. You et al. demonstrated air-stable PSCs with p-type NiO_x_ and n-type ZnO as HTL and ETL, respectively. The all-metal-oxide devices retain about 90% of the initial efficiency after 60 days of storage at room temperature (You et al., 2016). Fullerene-derived carbon materials such as PCBM and C60 have been developed as promising ETLs in inverted planar structures (Jeng et al., 2013). Besides, a broad range of metal oxides [ZnO (Liu and Kelly, 2014; Cheng et al., 2015), SnO_2_ (Jiang et al., 2016; Jiang et al., 2018), Zn_2_SnO_4_ (Shin et al., 2015) as ETLs and NiO_x_ (Jiang et al., 2015; Kim et al., 2015; Zhang et al., 2016), NiCo_2_O_4_ (Ouyang et al., 2018), CuGaO_2_ (Chen et al., 2018) as HTLs] are used in PSCs and deliver improved efficiency and stability.

**FIGURE 4:**
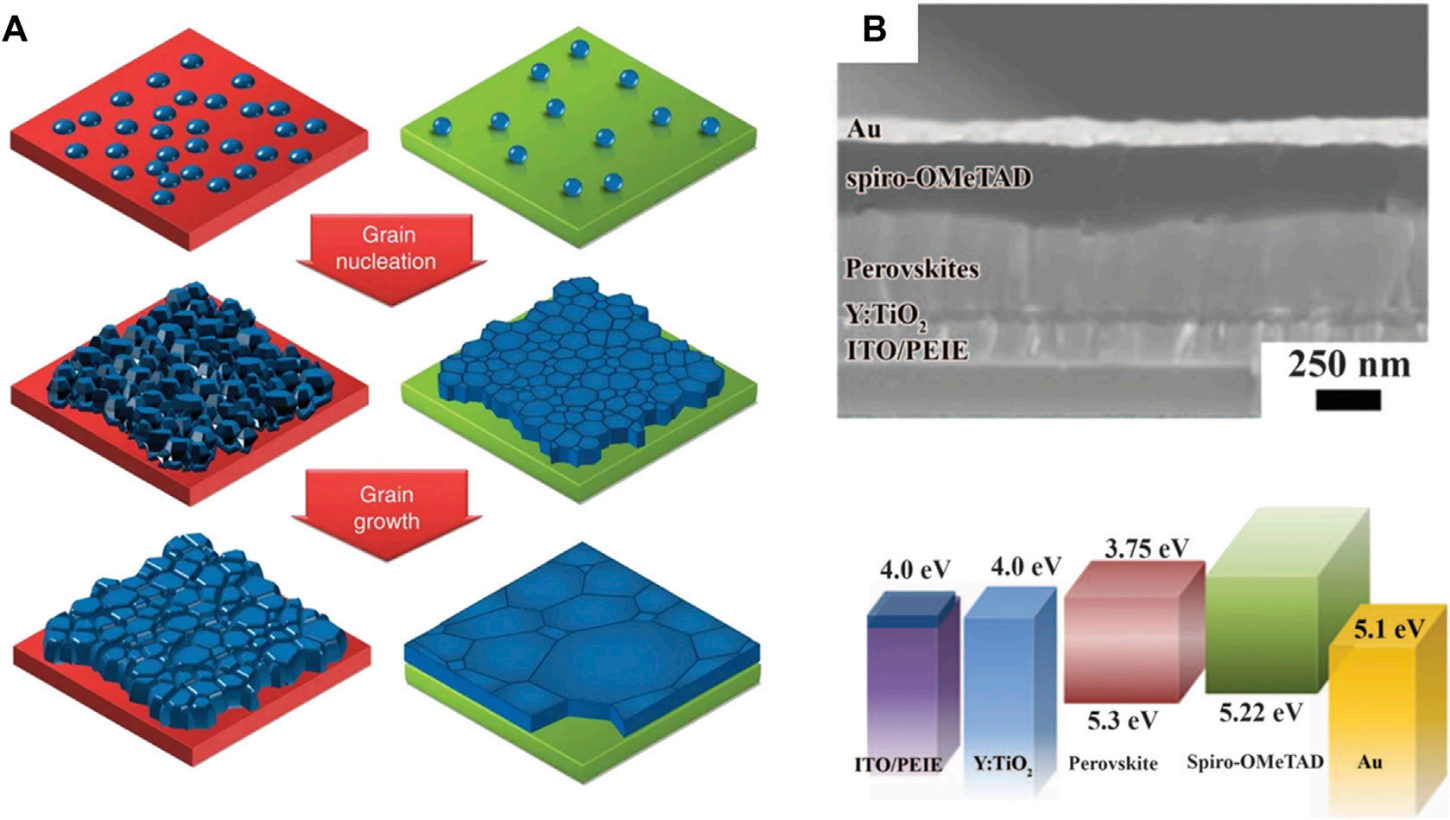
**(A)** The nucleation and growth of the grains on wetting and non-wetting surface. Adapted from Bi et al. (2015) with permission from Nature springer. **(B)** Device structure and energy levels of each functional layers in PSCs with PEIE interfacial modification. Reproduced from Zhou et al. (2014) with copyright from Science.

An important aspect of interfacial engineering is the impact of CTLs on perovskite formation. According to the Gibbs free energy [∆G_het = ∆G_hom × f(θ)], nucleation is determined by the interfacial free energy ∆G and wetting angle θ. Adjusting surface energy can control the nucleation and growth process of perovskite materials. Generally, hydrophilic CTLs lead to homogenous nucleation, resulting in perovskite films with high coverage and uniformity. On the contrary, hydrophobic surfaces reduce the nucleation, favoring the growth of large grain size. Huang et al. reported the growth of perovskite grains on non-wetting surfaces (Bi et al., 2015). The large-size perovskite grain featured a high grain size/thickness aspect ratio reaching 2.3–7.9, surpassing the limitation of the film thickness (Figure 4A).

The interfaces between CTLs and perovskite layers are crucial for PSCs, but imperfect with various trap states, which gives rise to intensive research on interfacial modifications. As early as 2014, Zhou et al. demonstrated that the electron extraction process was improved by modifying the interface with polyethyleneimine ethoxylated (Zhou et al., 2014), as shown in Figure 4B. These modifications led to a maximum cell efficiency of over 19%. Furthermore, deep trap states at the interfaces need to be suppressed to reduce interfacial non-radiative recombination loss. Chen et al. proposed that interfacial chlorine avoided the formation of deep delocalized states at the TiO_2_/perovskite interface (Tan et al., 2017). The interfacial recombination was mitigated by the contact passivation strategy. A similar effect was also observed on alkali chloride treated NiO_x_/perovskite interfaces that delivered improved ordering and reduced defect density (Chen et al., 2019b).

## Industrial Progress of PSCs

In 2022, the perovskite-on-silicon tandem solar cell hits the world-record efficiency of 29.5% in Oxford Photovoltaics (Oxford PV), which has been certified by National Renewable Energy Laboratory (NREL) (Al-Ashouri et al., 2020). This not only refreshes the record of 28.0% previously reported by this company but also breaks the performance record of 29.2% in the Helmholtz Zentrum Berlin (HZB). Instead of replacing silicon solar cells, the perovskite-on-silicon tandem configuration provides a more accessible strategy to develop PSCs in the existing photovoltaic market, which has been considered as very promising in the industrial process of PSCs. Oxford PV that was established in 2010 from the University of Oxford, is one of the first photovoltaic companies focusing on the commercialization of perovskite based photovoltaic technology. The mainstream perovskite-on-silicon tandem solar cell technology allows breaking the previous performance limit of cell modules. The perovskite-based tandem technology has shown great potential to be compatible with the current photovoltaic industry with predicted over 30% combined cell efficiency and the ease of integration.

At the same time, photovoltaic companies in China have been devoted to the scale-up production of PSCs. Hangzhou Microquanta Co. Ltd.’s firstly established the production line of 20 MW with 200–800 cm perovskite solar cell modules and achieved 11.98% PCE, breaking the previous world record held by Toshiba. In 2019, Suzhou GCL Nano Technology Co., Ltd. (GCL Nano), a subsidiary of GCL group, built a 10 MW production line for large-area perovskite modules, and completed the material synthesis and manufacturing process. Later, GCL announced to develop the product line of 100 MW production line and planned to realize the commercial production of perovskite modules. GCL Nano announced to achieve the PCE of 15.21% on an effective area of 1,241.16 cm^2^. Besides, the Jayu Group and its subsidiary Jayu Solar Energy Technology Co., Ltd. also pay considerable attention to the commercialization of the third-generation perovskite photovoltaic technology, and plan a new layout in their future production bases.

With the target of carbon neutrality in 2060, it is scheduled that non-fossil energy should account for 25% in 2030, and renewable energy capacity should increase to 1.2 billion KW or more. Next decade, the photovoltaic industry should reach 70–90 million KW per year compared with 50 million per year in the past 5 years.

## Merits and Future Challenges

Perovskite materials have advantages as photovoltaics such as light absorption coefficient up 10^4^ cm^−1^, long carrier diffusion length and solution processability. For example, MAPbI_3_ perovskites have covered a wide solar light range up to 800 nm with high light absorption coefficient. The use of FA^+^ cation extends the absorption edge to 840 nm, leading to remarkable photocurrent comparable to silicon solar cells (Jena et al., 2019; Min et al., 2019). The manufacturing of PSCs can be conducted through simple solution processes with low-cost earth-abundant elements such as C, H, Pb, I. It is estimated that the manufacturing cost of PSCs is only half of that of silicon solar cells (Meng et al., 2018). Compared with other commercial photovoltaic semiconductors, perovskites also exhibit higher defect tolerance (Ball and Petrozza, 2016), which delivers good reproducibility and reduces production requirements even in a module of 30 W. This merit is extremely desirable in the solution processes of films. In the large-scale integration, perovskite modules can adopt traditional rectangular photovoltaic modules that can be flexible, allowing to be intensively used on building roofs. Due to these advantages, the time horizon of PSCs is expected to be shorter than that of crystalline silicon photovoltaics.

However, there are still limitations that impede the commercialization of PSCs, for example, lead toxicity and long-term stability. Light-induced degradation of perovskites under operating conditions including moisture, oxygen, heat and current stress has become the major concern for outdoor applications of PSCs (Leijtens et al., 2015), even though crystal Si cells face similar problems when exposed to moisture and oxygen. However, the environmental impact can be resolved at the utmost by standard encapsulation technique. The future of PSCs should be bright in consideration of the history of copper indium gallium selenide (CIGS), which is first found to be unstable in the presence of water but has been widely used with long-term stability. Another instability issue is the use of organic interfacial layers such as PEDOT:PSS, Spiro-OMeTAD and so on. To solve these problems, many laboratories have been devoted to developing alternative inorganic interlayers with efficient carrier extraction. It is fortunate to see that, like the rapid development of cell efficiency, the long-term stability of PSCs has also increased a lot in the past several years. This can be attributed to the reduction in the intrinsic degradation of perovskites and the use of various CTLs with good stability. Nowadays, life-cycle assessment (LCA) of PSCs has reached more than 1,000 h (Industrial Standard for New Photovoltaic Technology), even as long as 10,000 h (Shi et al., 2020).

Another concern with perovskite-based photovoltaic technology is the toxicity of lead. Although it has been proposed that one of the main reasons for excellent optoelectronic properties in perovskite materials is the orbital overlap of Pb and I ions, the actual content of lead in PSCs is still lower than that in commercial modules. It is estimated that a perovskite cell module only contains 2 g of lead, while the value of a crystalline silicon cell module is 16 g. Furthermore, encapsulation and recycling techniques have been developed to prevent lead pollution. Obviously, the use of lead inevitably causes the risk of environmental pollution and increases the additional cost in the recycling process. Scientists have always been looking for ideal elements to replace lead in perovskite materials. Tin (Sn) is one of the potential elements to avoid the use of lead, but it is companied by the instability issue because of the oxidation of Sn^2+^ to Sn^4+^.

## Future Prospects of the Commercialization

In the past several years, the manufacturing costs of silicon solar cells have dropped significantly that makes it possible to use photovoltaics as the large-scale clean energy supply to replace the fossil fuels. Many studies suggest that the solar energy can completely compete with conventional plants when the cost is below than 32 cents/W in well-equipped regions with sufficient solar light. However, the silicon based photovoltaic technology has approached its cost-effective limit due to thermalization loss, low absorption coefficient, and high material requirements. PSCs provides opportunities to go well beyond silicon based photovoltaic technology. Compare with silicon based photovoltaic technology that requires highly pure raw materials (99.999%), PSCs can be manufactured with relatively low purity (> 99%). More importantly, perovskite films are fabricated by low temperature solution routes that result into less power assumption and environmental pollution. Although the second-generation gallium arsenide thin-film solar cell has higher PCEs of about 30%, the cost is extremely high. It is expected that the manufacturing cost of perovskite modules would be 50% lower than that of monocrystalline silicon. However, it is difficult to displace silicon solar cells in photovoltaic market due to the lack of demonstrated production line. Alternatively, the perovskite-on-silicon tandem configuration should be one of promising technological routes in industrial progress of PSCs. The ease of tuning the bandgap of perovskites and compatible processes make them ideal wide-bandgap materials in the silicon based multi-junction solar cells, although there are some challenges in the transparent conductive layers, tunnel junctions, and light utilization (Li and Zhang, 2020). In the commercialization of PSCs, the supply chain for manufacturing is shorter, and large-scale coating equipment can be developed rapidly. The key technologies for large-area flexible perovskite solar photovoltaic cells will be achieved in about 3 years. The pathways of PSCs from the laboratory to market are emerging with numerous the efforts in academic and industrial field. The commercialization of PSCs is estimated to give rise to at least 100 billion in the photovoltaic market.
