# Erratum: A perspective on perovskite solar cells: emergence, progress, and commercialization

**DOI:** 10.3389/fchem.2025.1562415

**Published:** 2025-01-31

**Authors:** Frontiers Production Office

**Affiliations:** Frontiers Media SA, Lausanne, Switzerland

**Keywords:** perovskite, solar cells, composition engineering, process engineering, interfacial engineering, industrial progress, commercialization

Following publication, we found a non-genuine email address had been provided for reviewer Alaa El-Din Bekhit. Following an investigation, which was conducted in accordance with Frontiers policy, we confirmed that the real Alaa El-Din Bekhit was impersonated and did not take any action on this manuscript and has therefore been removed from this article. In line with the COPE guidelines on potential peer review manipulation, we conducted a post-publication assessment that concluded that the article meets the standards for publication at Frontiers.

